# Safety and efficacy of chidamide for maintenance therapy after allogeneic hematopoietic stem cell transplantation in patients with T-ALL/T-LBL

**DOI:** 10.1007/s00277-026-07016-x

**Published:** 2026-05-09

**Authors:** Kuangfei Wang, Xiaojing Li, Xiaofan Li, Xiaohong Yuan, Xianling Chen, Xiaohui Lai, Nainong Li, Ping Chen

**Affiliations:** Fujian Provincial Key Laboratory on Hematology, Fujian Institute of Hematology, Fujian Medical University Union Hospital, Fuzhou, Fujian China

**Keywords:** Acute T-lymphoblastic leukemia, Histone deacetylase inhibitor, Chidamide, Allogeneic hematopoietic stem cell transplantation, Maintenance therapy, Efficacy

## Abstract

Post-transplant relapse remains a primary obstacle to long-term survival in patients with T-cell acute lymphoblastic leukemia/lymphoma (T-ALL/T-LBL), underscoring the need for effective maintenance therapies. Although chidamide, a selective histone deacetylase inhibitor, has shown activity in T-cell malignancies, its role as post-transplant maintenance has been scarcely studied. To evaluate the efficacy and safety of chidamide as maintenance therapy in T-ALL/T-LBL patients after allogeneic hematopoietic stem cell transplantation (allo-HSCT). We retrospectively analyzed 75 T-ALL/T-LBL patients who underwent allo-HSCT (December 2019–January 2024). Thirty-eight patients received chidamide-based maintenance, and 37 served as controls (no maintenance or non-chidamide regimens). The primary endpoints were overall survival (OS) and progression-free survival (PFS); safety endpoints included graft-versus-host disease (GVHD) incidence and treatment-related adverse events. Chidamide maintenance was associated with significantly improved OS and PFS compared with the non-chidamide group (OS: *p* = 0.001; PFS: *p* = 0.006). Multivariate Cox regression identified chidamide maintenance (OS: *p* = 0.003; PFS: *p* = 0.006) and complete remission status before transplantation (OS: *p* = 0.013; PFS: *p* = 0.012) as independent favorable prognostic factors. Chidamide did not increase acute GVHD; chronic GVHD was numerically higher (26.32% vs. 13.51%) but mostly mild to moderate and manageable. Adverse events were primarily hematologic toxicity (bone marrow suppression) and gastrointestinal, generally manageable and rarely required treatment discontinuation. Chidamide maintenance after allo-HSCT significantly improves survival outcomes in T-ALL/T-LBL patients with an acceptable safety profile, while pre-transplant remission status further refines prognosis. Prospective studies are warranted to confirm these findings and optimize maintenance strategies in this setting.

## Introduction

T-cell acute lymphoblastic leukemia (T-ALL) and T-cell lymphoblastic lymphoma (T-LBL) are considered the same disease with different clinical manifestations and stages of development. These are highly invasive tumors originating from immature precursor T lymphocytes, characterized by high malignancy and significant heterogeneity [1]. The rate of complete remission (CR) is low, with a median survival of only 11–17 months [2]. Currently, induction therapy based on risk stratification, utilizing intensive multi-drug combination chemotherapy regimens (including CALLG-2008, GRAALL-2005, CALGB8811, and hyper-CVAD, among others [3]), followed by allogeneic hematopoietic stem cell transplantation (allo-HSCT), is the primary treatment for T-ALL/T-LBL. Allo-HSCT is the only curative option for these diseases. However, disease recurrence post-transplantation remains a major cause of treatment failure. Increasing evidence indicates that residual malignant cells often persist after HSCT, particularly in the bone marrow, which is a key factor contributing to disease relapse. How can residual malignant cells be effectively eliminated? Beyond relying on the graft-versus-leukemia (GVL) effect of hematopoietic stem cells, maintenance therapy after transplantation is critical. Its goal is to reduce the risk of recurrence by targeting residual malignant cells, thereby prolonging the remission period and improving disease prognosis. Nonetheless, previous studies have shown that PFS and OS in patients receiving maintenance therapy after transplantation have not been significantly extended, likely due to the cytotoxic chemotherapy drugs commonly used in maintenance regimens [4]. In recent years, novel agents such as demethylating drugs and Bcl-2 inhibitors have shown promising results in the maintenance treatment of MDS and AML following transplantation. Studies by Feng [5] and Marcos [6] have demonstrated that the use of low-dose demethylating agents for maintenance therapy after allo-HSCT in AML patients can effectively prevent relapse without increasing the risk of GVHD, infections, or other adverse events.

Histone modification is a crucial epigenetic regulation mechanism. Histone deacetylases (HDACs) are dysregulated in diverse tumors. Histone deacetylase inhibitors (HDACi) target HDACs to elevate histone acetylation, induce chromatin remodeling, and regulate gene expression, thereby exerting antitumor effects. Six HDAC inhibitors are currently FDA-approved for the treatment of hematologic malignancies and solid tumors [7]. Chidamide, a selective HDACi from the benzamide class, has shown promise in the treatment of T-ALL. Research has confirmed that chidamide can induce necrotic apoptosis in T-ALL cells, which is associated with mitochondrial damage, down-regulation of c-FLIPL, and activation of caspase and RIP3/MLKL signaling pathways [8]. Other studies have demonstrated that chidamide can enhance the anti-leukemia effects of chemotherapy drugs in ALL [9]. Furthermore, chidamide also has regulatory effects, such as reducing pro-inflammatory factor levels and enhancing the function of donor regulatory T cells and NK cells, without significantly impairing anti-leukemia activity, thereby reducing the incidence of GVHD [10]. As a result, chidamide could be an effective agent in maintenance therapy.

This paper presents a retrospective analysis of the clinical data on chidamide maintenance therapy in T-ALL/T-LBL patients after allo-HSCT. The analysis examines remission rates, treatment-related adverse reactions, and other relevant indicators. The aim is to evaluate the efficacy and safety of chidamide monotherapy or chidamide in combination with other drugs as maintenance therapy in T-ALL/T-LBL patients post-transplant, providing a clinical basis for selecting an appropriate maintenance treatment plan.

## Materials and methods

### Objects of study

Clinical data and treatment outcomes were collected from 75 patients with T-ALL or T-LBL who underwent transplantation at our hospital between December 2019 and January 2024. Thirty-eight patients received chidamide maintenance therapy, while 37 patients either did not receive chidamide or any maintenance therapy. Inclusion criteria: ① The diagnosis was confirmed according to the Chinese Guidelines for the Diagnosis and Treatment of Adult Acute Lymphoblastic Leukemia (2021 edition) prior to transplantation; ② Patients in the chidamide group were treated with chidamide for at least one month post-transplantation. The efficacy of T-ALL was evaluated based on the National Comprehensive Cancer Network (NCCN) guidelines and the Chinese Guidelines for the Diagnosis and Treatment of Adult Acute Lymphoblastic Leukemia (2021 edition).

### Treatment

All patients underwent allo-HSCT. Patients in the control group either did not receive any maintenance therapy post-transplant or did not have chidamide included in their maintenance regimen. Patients in the chidamide group were treated with chidamide maintenance therapy, which included either chidamide monotherapy or chidamide combined with hypomethylatingagents, interferon, or other drugs. Chidamide tablets were orally administered at a dose of 30 mg per session after breakfast, twice a week, depending on patient tolerance. When grade 3 or 4 neutropenia (< 1.0 × 10^9^/L) or thrombocytopenia (< 50 × 10^9^/L) occurred, chidamide administration was paused until the absolute neutrophil count recovered to ≥ 1.5 × 10^9^/L and the platelet count recovered to ≥75 × 10^9^/L. After two consecutive tests confirming these recovery levels, chidamide treatment was resumed. All 38 patients in the chidamide group were treated for more than one month, and a short-term efficacy evaluation was conducted at six months.

### Observed indexes

Clinical characteristics of patients meeting the inclusion criteria were collected, including gender and age. Laboratory data included routine blood tests, liver and kidney function, and bone marrow cytology with lymphoblast and prolymphocyte counts. Additional assessments included flow immunophenotyping, minimal residual disease, gene mutations, chromosome analysis, the course of enhanced consolidation therapy, transplantation method, post-transplant complications, and other relevant indicators. Some of these indicators were monitored during follow-up throughout treatment.

### Adverse reaction evaluation

Adverse reactions were assessed according to the WHO chemotherapeutic drug adverse reaction grading standard (version 3.0).

### Statistical method analysis

Statistical analysis was conducted using GraphPad Prism 9.5, SPSS 27.0, and R 4.2.3. Measurement data were expressed as mean ± SD or median. The chi-square test was used to compare categorical data between groups. When the total sample size was less than 40 or the minimum theoretical frequency was less than 1, Fisher’s exact probability test was employed. The log-rank test was used to assess OS and PFS. Prognostic indicators were identified through Lasso regression, collinearity analysis, and Cox regression analysis. A test level of 0.05 was used, with *p* < 0.05 considered statistically significant.

## Statistical result

### Patient baseline characteristics

This study included 75 patients with T-ALL or T-LBL, comprising 43 T-ALL patients and 32 T-LBL patients. Among these, 38 patients received maintenance therapy with chidamide, while 37 either did not receive chidamide or had no maintenance therapy. Based on our study records, among the 37 patients in the non-chidamide group, 31 received no maintenance therapy, while 6 received other maintenance regimens (interferon, *n* = 4; hypomethylating agents [HMAs], *n* = 2). Further details are provided in Table 1.

**Table 1:** Baseline characteristics of 75 patients with T-ALL/T-LBL after allo-HSCT

Patients'characteristics		Non-Chidamide group	Chidamide group	*P*
Number of cases		37	38	
Diagnosis	T-ALL	24	19	0.245
	T-LBL	13	19	
Gender				
	Male	28	25	0.347
	Female	9	13	
Age				
	≤23	19	19	0.907
	>23	18	19	
WBC acount (×10^9^/L )				
	≤10	22	22	0.891
	>10	15	16	
CR before transplantation				
	Yes	26	30	0.388
	No	11	8	
Extramedullary infiltration				
	Yes	22	29	0.118
	No	15	9	
CNS involvement				
	Yes	3	6	0.480
	No	34	32	
Granulocyte implantation time ( days )				
	≤15	21	27	0.197
	>15	16	11	
Platelet implantation time ( days )				
	≤21	21	17	0.298
	>21	16	21	
Pulmonary infection after transplantation				
	Yes	24	21	0.482
	No	13	17	
Strengthen course of treatment				
	≤4 times	34	30	0.191
	>4 times	3	8	
aGVHD after transplantation				
	Yes	14	10	0.329
	No	23	28	

### The clinical efficacy of chidamide maintenance therapy in T-ALL/T-LBL patients after transplantation

Among the 75 patients, the 1-year, 3-year, and 5-year OS rates were 72.58%, 60.79%, and 60.79%, respectively. The 1-year, 3-year, and 5-year PFS rates were 64.29%, 55.55%, and 55.55%, respectively (OS: Fig. 2a; PFS: Fig. 2b). The log-rank test showed that patients with chidamide maintenance therapy had significantly higher OS and PFS compared to those without chidamide or no maintenance therapy. This difference was statistically significant (OS: *p* = 0.001, Fig. 2a; PFS: *p* = 0.006, Fig. 2b). To mitigate immortal time bias, we applied landmark analyses to accurately compare patients receiving and not receiving chidamide therapy. This reduced the overestimation of treatment efficacy caused by differences in follow-up time. Patients were grouped by whether they started chidamide maintenance therapy at predetermined time points (6, 9, 12, 15, 18, and 24 months post-transplantation). Landmark sensitivity analyses were conducted at these time points to test the robustness. Findings showed that the timing of chidamide initiation did not significantly affect OS benefit (Table 2). 

**Fig. 1:**
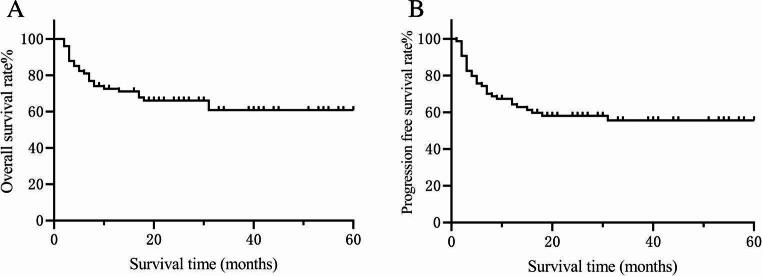
Overall survival and progression-free survival in 75 patients

**Fig. 2:**
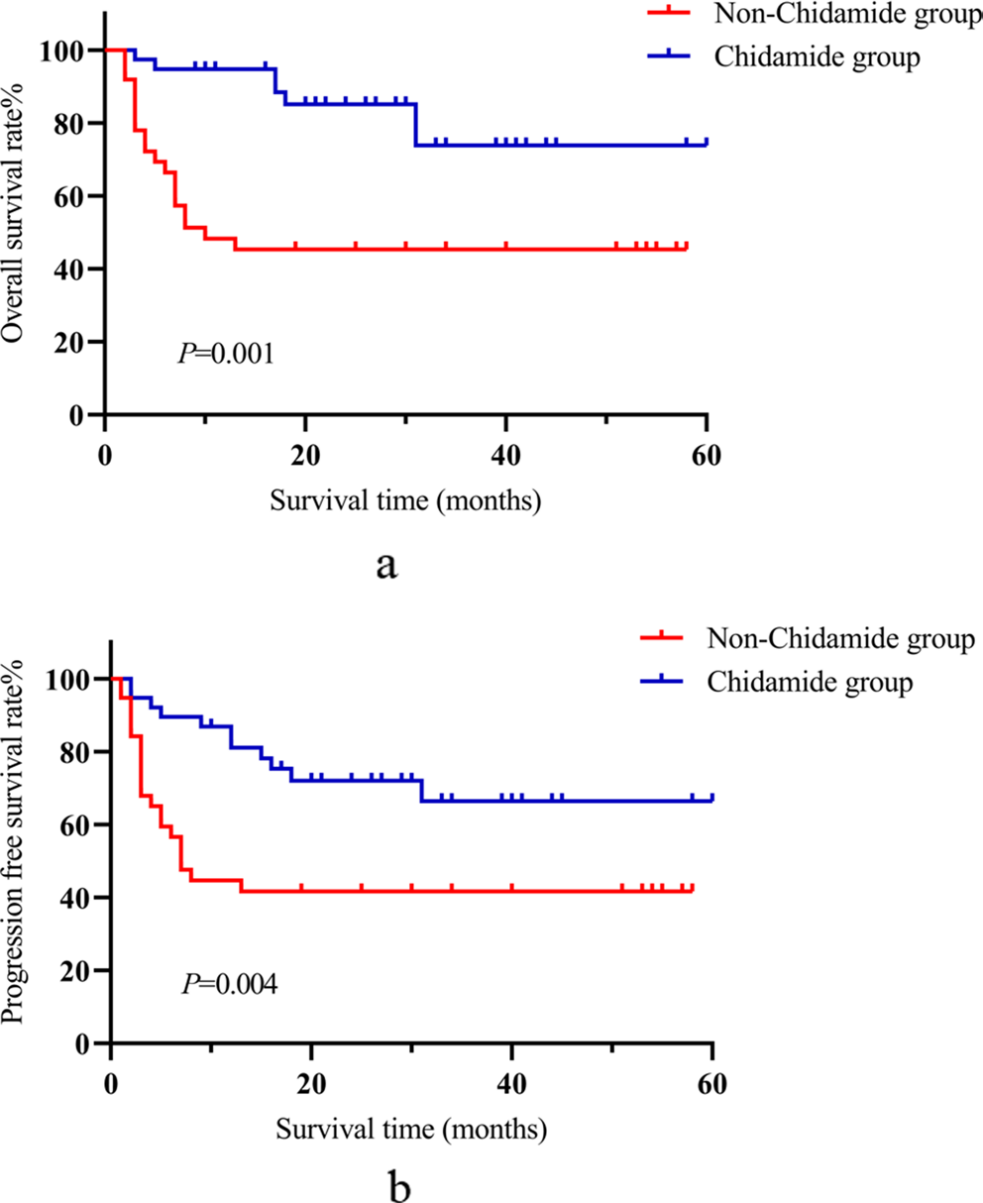
Comparison of OS and PFS between chidamide group and non-chidamide group

**Table 2:** Landmark analysis of maintenance therapy with chidamide at different time periods after transplantation

Landmark time months	Pts on treatment prior to landmark time, *n*	Whether to use chidamide prior to landmark time	Subgroup, *n*	*P*
6	62 (82.67%)	Yes	25	0.0058
		No	37	
9	62 (82.67%)	Yes	25	0.0058
		No	37	
12	68 (90.67%)	Yes	31	0.0055
		No	37	
15	71 (94.67%)	Yes	34	0.0020
		No	37	
18	74 (98.67%)	Yes	37	0.0007
		No	37	
24	75 (100%)	Yes	38	0.0005
		No	37	

Yes for maintenance therapy with Chidamide, no for maintenance therapy without Chidamide; The same patients met landmark inclusion criteria at both 6 and 9 months, resulting in identical patient counts and *p*-values

**Fig. 3:**
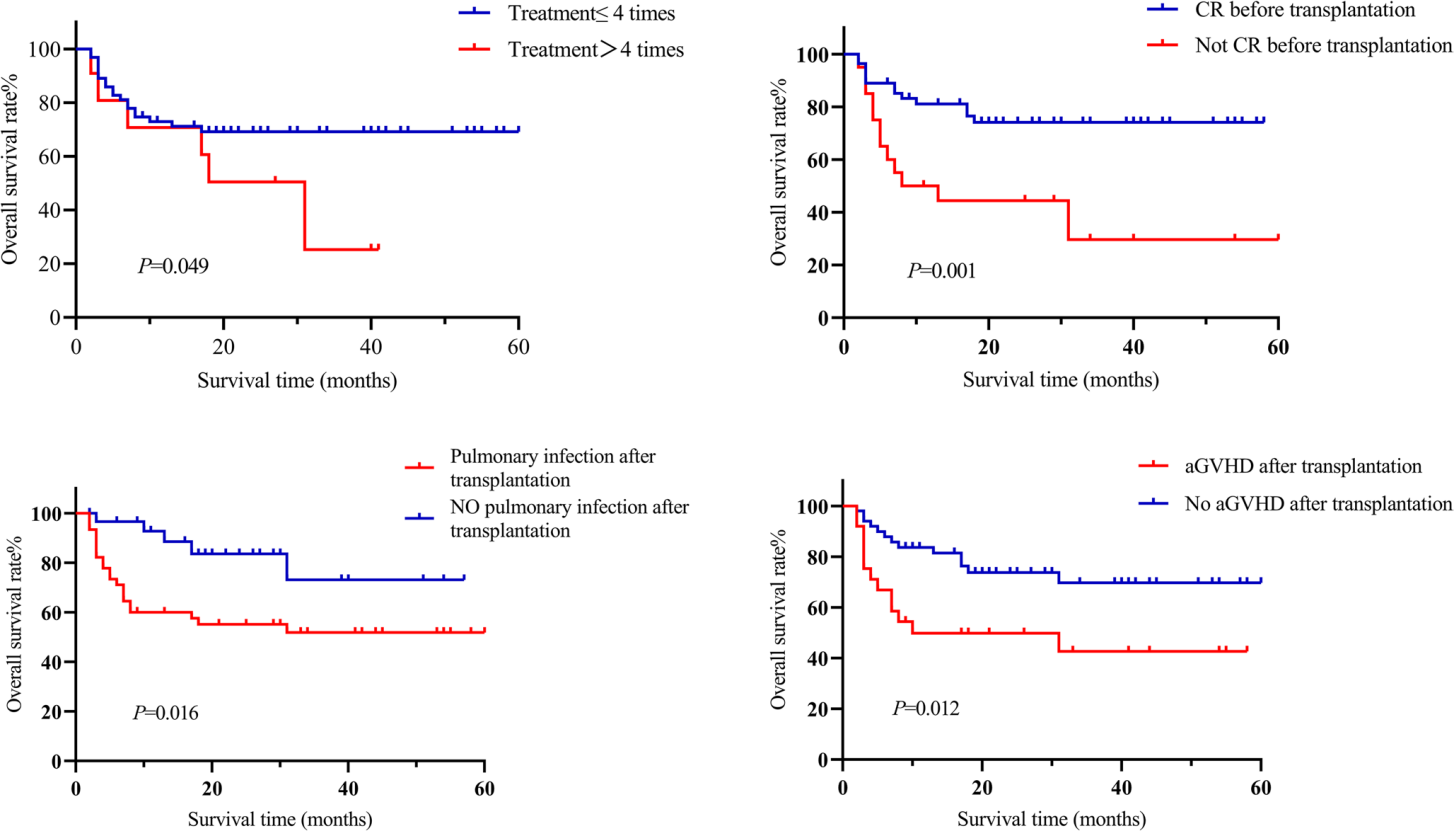
Factors affecting OS in 75 patients

Lasso regression analysis was used to identify factors strongly associated with prognosis. Twelve factors, including age, gender, white blood cell count at diagnosis, extramedullary lesions, CNS involvement, disease status before transplantation, intensive consolidation course prior to transplantation, granulocyte engraftment time, platelet engraftment time, post-transplant pulmonary infection, aGVHD after transplantation, and chidamide maintenance therapy, were analyzed to assess their impact on OS and PFS in the T-ALL/T-LBL patients. The final variables included in the model were disease status before transplantation, course of intensive consolidation therapy, granulocyte engraftment time, post-transplant pulmonary infection, chidamide maintenance therapy, and post-transplant aGVHD. Univariate analysis using the log-rank test revealed that intensive consolidation therapy (*p* = 0.049), disease status before transplantation (*p* = 0.001), post-transplant pulmonary infection (*p* = 0.016), post-transplant aGVHD (*p* = 0.012), and chidamide maintenance therapy (*p* = 0.001) could be factors affecting OS (Fig. 3).

**Fig. 4:**
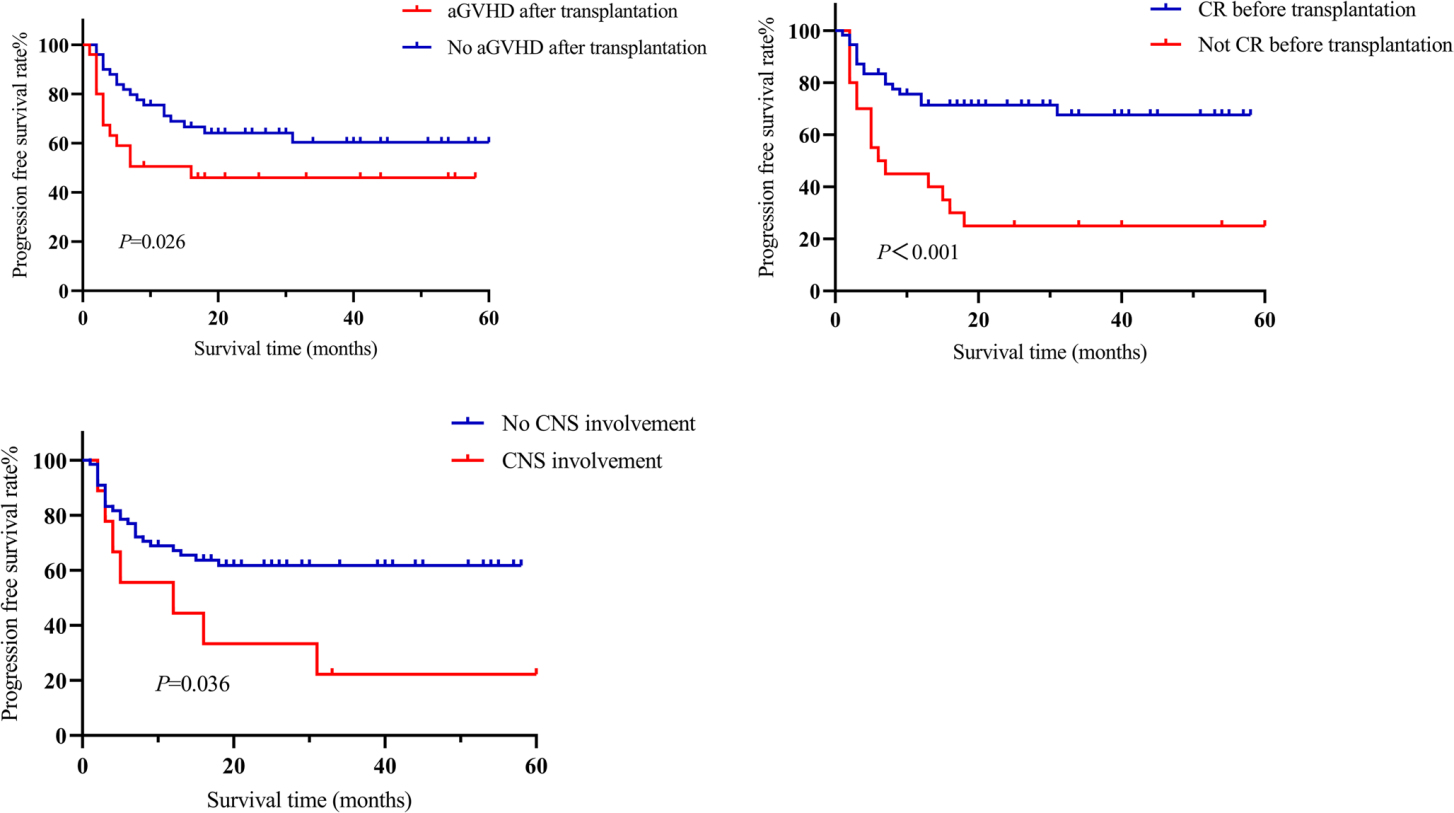
Factors affecting PFS in 75 patients

Central nervous system (CNS) involvement (*p* = 0.036), disease remission status before transplantation (*p* < 0.001), and aGVHD after transplantation (*p* = 0.026) were associated with PFS (Fig. 4).

Univariate and multivariate Cox regression analysis demonstrated that chidamide maintenance therapy was independently associated with significant improvements in OS and PFS in post-transplant T-ALL/T-LBL patients. Additionally, pre-transplant CR status and absence of aGVHD were identified as independent protective factors for OS (pre-transplant CR: *p* = 0.013; absence of aGVHD: *p* = 0.049; chidamide maintenance: *p* = 0.003; Table 3).

Chidamide maintenance therapy (*p* = 0.006) and pre-transplant disease status (*p* = 0.012) were independent prognostic factors for PFS in T-ALL/T-LBL patients. Patients with pre-transplant complete remission and post-transplant chidamide maintenance therapy had a better prognosis (Table 4).

**Table 3:** Univariate and multivariate COX analysis of OS in 75 patients

	Univariate COX analysis			Multivariate COX analysis		
	HR	95% CI	*P*	HR	95% CI	*P*
Disease remission status before transplantation	3.295	1.532–7.129	0.002	2.748	1.239–6.093	0.013
Whether to use Chidamide	0.246	0.103–0.589	0.002	0.261	0.108–0.632	0.003
Whether aGVHD occurs	2.544	1.177–5.499	0.018	2.214	1.001–4.897	0.049

**Table 4:** Univariate and multivariate COX analysis of PFS in 75 patients

	Univariate COX analysis			Multivariate COX analysis		
	HR	95% CI	*P*	HR	95% CI	*P*
CNS involvement	2.362	1.017–5.489	0.046	2.515	0.996–6.352	0.051
Disease remission status before transplantation	3.269	1.611–6.636	0.001	2.61	1.238–5.501	0.012
Whether to use Chidamide	0.376	0.179–0.790	0.01	0.333	0.152–0.731	0.006

**Fig. 5:**
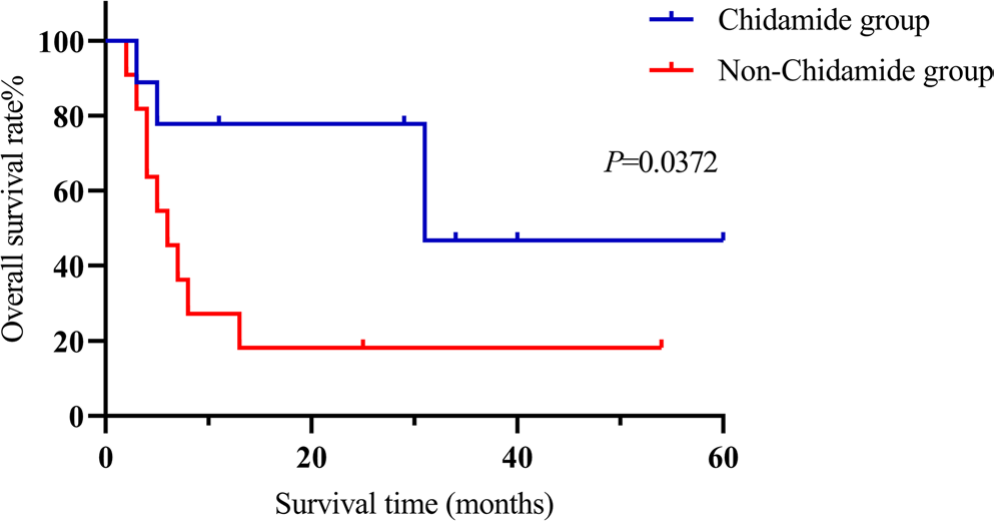
OS of patients in the chidamide group versus the non-chidamide group in the non-CR state before transplantation

**Fig. 6:**
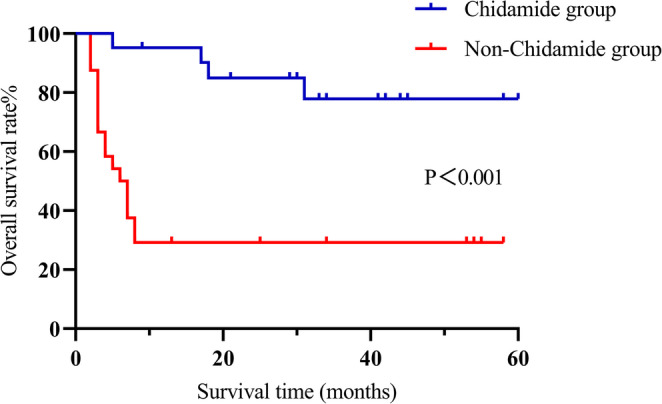
OS of patients in the chidamide group versus the non-chidamide group who developed pulmonary infection after transplantation

**Fig. 7:**
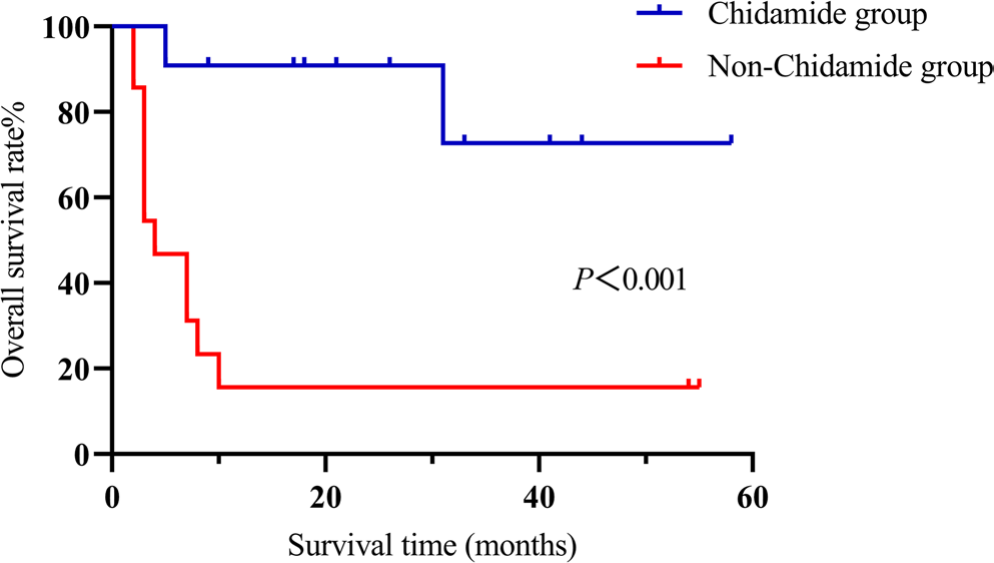
OS of patients in the chidamide group versus the non-chidamide group who developed aGVHD after transplantation

To further evaluate chidamide’s effect on post-transplant prognosis in high-risk T-ALL/T-LBL patients, a log-rank test was performed based on known risk factors. The results demonstrated that chidamide-containing maintenance therapy led to better clinical outcomes across different risk stratifications, confirming its beneficial role in improving prognosis (Figs. 5, 6 and 7).

### Chidamide alone vs. combination maintenance therapy 

**Fig. 8:**
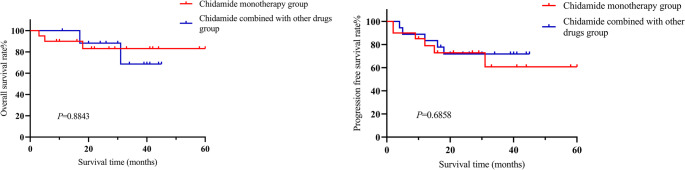
OS and PFS in the chidamide monotherapy group vs. the combination group

Patients receiving chidamide maintenance therapy post-transplant were categorized into two intervention groups: chidamide monotherapy or combination therapy with hypomethylating agents (HMAs) and interferon. The detailed treatment protocols are summarized in Table 5. Comparative survival analysis using the Kaplan-Meier method between the monotherapy and combination therapy groups revealed that no significant differences were observed in OS and PFS between the two groups (OS: *p* = 0.8843; PFS: *p* = 0.6858) (Fig. 8 ).

**Table 5:** Nature and schedule of drugs used for maintenance

Medication	Median time to drug initiation (months post-transplant)	Maintenance duration (months)	Dosage and frequency
Chidamide	5 ( 2–19 )	12 ( 3–24 )	30 mg, twice weekly
HMA	6 ( 3–12 )	4 ( 2–6 )	5 mg/m^2^, days 1–5
Interferon	6.5 ( 3–13 )	6 ( 3–8 )	3 MIU, twice weekly

### Chidamide vs. non-chidamide group for GVHD occurrence

Among 38 T-ALL/T-LBL patients receiving post-transplant chidamide maintenance, 10 developed aGVHD, including 8 (21.05%) with grade I–II and 2 with grade III–IV aGVHD; 10 patients (26.32%) developed cGVHD, of whom 9 had mild and 1 had moderate-to-severe cGVHD; all cases were manageable with immunosuppressive therapy without requiring chidamide discontinuation. In the non-chidamide group (*n* = 37), aGVHD occurred in 14 patients (3 [8.1%] grade I–II and 11 [29.73%] grade III–IV); cGVHD was reported in 5 (13.51%). Comparative analysis indicated a lower incidence and severity of aGVHD in the chidamide group (Table 6).

**Table 6:** Occurrence of GVHD in T-ALL/T-LBL patients after transplantation

	Grade	Non-Chidamide group	Chidamide group
aGVHD	Grade I-II	3	8
	Grade III-IV	11	2
cGVHD	Mild	1	9
	Moderate/Severe	4	1

### Adverse reactions of chidamide maintenance therapy

In this study, among the 38 T-ALL/T-LBL patients who received chidamide maintenance therapy post-transplant, 36 experienced bone marrow suppression. Of these, 9 patients (25%) had grade I, 15 (41.67%) had grade II, 10 (27.78%) had grade III, and 2 (5.55%) had grade IV. The number of patients with varying levels of bone marrow suppression and the corresponding minimum ranges are presented in Table 7.

**Table 7:** Hematologic adverse reactions during treatment with chidamide

	WBC(*10^9^/L)	NEUT(*10^9^/L)	HB(g/L)	PLT(*10^9^/L)
Grade 0	19(4.07-8.76)	7(2.20-4.94)	22(111-162)	21(100-195)
GradeⅠ	11(3-3.88)	9(1.58-1.99)	9(95-105)	8(75-92)
GradeⅡ	7(2.00-2.65)	12(1.06-1.47)	4(83-93)	9(50-74)
GradeⅢ-Ⅳ	1(1.45)	10(0.43-0.94)	3(62-74)	0

**Fig. 9:**
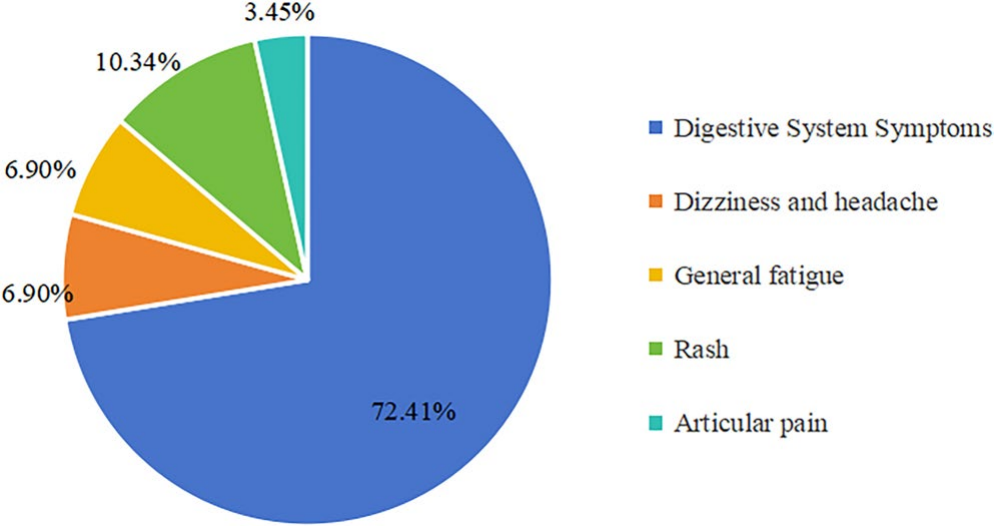
Non-hematologic adverse reactions of chidamide

After chidamide maintenance therapy, the most common non-hematological adverse reactions were gastrointestinal, occurring primarily on the day of medication and the following day. A few patients experienced dizziness, general fatigue, rash, joint pain, and other side effects. Most patients tolerated these adverse reactions after receiving symptomatic and supportive treatment (Fig. 9). No adverse reactions related to renal toxicity, cardiac toxicity, or serous cavity effusion were observed.

## Discussion

Acute T-cell lymphoblastic leukemia (T-ALL) and T-cell lymphoblastic lymphoma (T-LBL) are both derived from T-cell lineage lymphoblasts and share similarities in cell morphology, immunophenotype, genotype, cytogenetics, clinical presentation, and prognosis. Consequently, the World Health Organization and the International Lymphoma Study Group now classify T-ALL and T-LBL as the same disease in the current WHO and Western lymphoma classifications [11]. Current treatment for T-ALL/T-LBL mainly consists of multi-drug induction regimens, consolidation therapy, and maintenance therapy. For eligible patients, hematopoietic stem cell transplantation is recommended. Allogeneic hematopoietic stem cell transplantation (allo-HSCT) can improve overall survival in T-ALL/T-LBL patients; however, disease relapse post-transplantation remains the leading cause of treatment failure. Post-transplant relapse occurs in 20–60% of T-ALL/T-LBL patients, with approximately 46% dying within one year after relapse. The prognosis is poor, with a median DFS of only 6.9 months. The 1‑, 2‑, and 5‑year survival rates after relapse were 30 ± 2%, 16 ± 2%, and 8 ± 1%, respectively [12].

Recurrence after transplantation remains the central issue limiting the quality of life for T-ALL/T-LBL patients. Multiple studies have confirmed that residual malignant cells, particularly within the bone marrow microenvironment, are a major cause of relapse. Therefore, in addition to utilizing the graft-versus-leukemia (GVL) effect, post-transplant maintenance therapy is increasingly emphasized. Recent advances in novel agents, including hypomethylating agents and Bcl-2 inhibitors, offer new promise for this strategy. Histone deacetylase inhibitors (HDACi) exert anti-tumor effects by inhibiting histone deacetylases, enhancing acetylation, and regulating gene expression to induce cell cycle arrest or apoptosis through multiple anti-tumor pathways [13, 14]. Selective HDACi have been shown to effectively inhibit the proliferation of T-ALL cell lines A3 and Molt-4 and to induce apoptosis through specific mitochondrial pathways [15]. This mechanism is thought to be associated with the inhibition of the ERK/MAPK signaling pathway. Endogenously developed in China, it selectively inhibits subtypes 1, 2, and 3 of class I histone deacetylases (HDAC) and subtype 10 of class IIb [16]. It guides tumor cell differentiation and apoptosis by regulating epigenetic mechanisms. A domestic trial involving patients with R/R T-ALL/LBL demonstrated that the group receiving chidamide in combination with chemotherapy had improved progression-free survival (PFS) compared with the chemotherapy-only group. A clinical trial conducted at Ruijin Hospital in newly diagnosed or relapsed T-ALL patients revealed that chidamide reduced minimal residual disease (MRD) in patients with NOTCH1 mutations and was well tolerated. This suggests that chidamide offers a safe and effective treatment option for T-ALL patients with NOTCH1 mutations, providing a promising new therapeutic strategy [17].

There are limited studies on the use of chidamide as maintenance therapy after allo-HSCT in T-ALL/T-LBL patients. This paper retrospectively analyzed clinical data from T-ALL/T-LBL patients who received chidamide maintenance therapy post-allo-HSCT and compared them with those who did not receive chidamide maintenance. The objective was to evaluate the efficacy and safety of chidamide as a maintenance therapy in combination with multi-drug chemotherapy. Patients receiving chidamide maintenance had significantly higher OS (*p* = 0.001) and PFS (*p* = 0.006) than the non-chidamide group. Multivariate Cox regression of 75 post-transplant T-ALL/T-LBL patients showed that chidamide maintenance therapy (OS: *p* = 0.003; PFS: *p* = 0.006), pre-transplant disease status (OS: *p* = 0.013; PFS: *p* = 0.012), and post-transplant aGVHD (OS: *p*= 0.049) were independent prognostic factors for survival, suggesting a potential benefit of chidamide in improving outcomes.

This result suggests that when transplant conditions are favorable for T-ALL / T-LBL patients, transplantation should be performed as soon as possible when the disease is in CR or MRD-negative. The use of chidamide to maintain PFS or OS in T-ALL/T-LBL patients post-transplant is more effective than without chidamide or in non-maintenance groups, thereby reducing the risk of recurrence. Further analysis of 38 patients receiving chidamide-containing regimens showed similar outcomes between monotherapy and combination therapy. Therefore, chidamide monotherapy can be considered a viable maintenance option for T-ALL/T-LBL patients post-transplant. Patients receiving chidamide had a lower proportion of severe aGVHD (grade III–IV) compared to the non-chidamide group. Regarding cGVHD, although the chidamide group showed a higher cumulative incidence, the majority of cases were mild and did not necessitate treatment interruption. Common adverse reactions included hematological effects, primarily bone marrow suppression, such as decreased neutrophil counts, hemoglobin levels, and platelet counts in peripheral blood. Non-hematological adverse reactions were mainly gastrointestinal, including nausea and vomiting, with a few patients reporting dizziness, general fatigue, and rashes. Most patients tolerated these side effects with appropriate symptomatic and supportive care. In conclusion, our findings indicate that chidamide maintenance therapy is associated with improved survival after allo-HSCT in patients with T-ALL/T-LBL and exhibits a manageable safety profile. It should be noted that the control group in this study included patients receiving no maintenance (*n* = 31) or other maintenance therapies (interferon/HMAs, *n* = 6). However, the subgroup receiving other maintenance therapies is both very small and highly heterogeneous (interferon vs. HMAs differ in their mechanisms and clinical use patterns). Therefore, any statistical comparison would be substantially underpowered and could produce unstable or potentially misleading estimates. For this reason, we retained the primary analysis comparing chidamide maintenance versus the overall non-chidamide control group, which is the most interpretable approach given the available data. Furthermore, this study has several limitations. First, its retrospective, single-center design inherently introduces selection bias and limits causal inference. In particular, allocation to the chidamide versus non-chidamide group was non-randomized and based on real-world physician-directed decision-making rather than a prespecified protocol. Although baseline characteristics were broadly comparable between groups for measured variables (Table 1), residual confounding cannot be excluded. Second, the clinical criteria influencing the initiation of chidamide maintenance were not standardized. In routine practice, chidamide was generally considered for patients who achieved complete remission, had adequate hematopoietic recovery, and lacked uncontrolled severe infection or active severe acute GVHD, and it was more likely to be recommended for patients perceived to have higher relapse risk (e.g., MRD concern). Such a clinician’s judgment may have introduced confounding by indication. Third, several potentially important determinants of treatment accessibility and tolerability were not systematically captured, including ECOG performance status and socioeconomic/financial factors (e.g., insurance coverage and out-of-pocket affordability). Because these data were unavailable, we were unable to adjust for their influence on treatment assignment and outcomes. Finally, the sample size was modest, and follow-up duration varied, which may reduce statistical power for subgroup analyses and for detecting less common adverse events. Despite these limitations, our findings provide important preliminary evidence suggesting that chidamide maintenance is associated with improved survival outcomes and a manageable safety profile in T-ALL/T-LBL patients after allo-HSCT. Collectively, these results underscore the potential of chidamide in this setting and strongly justify the need for larger, prospective, and ideally randomized controlled trials to definitively establish its efficacy, optimize patient selection, and rigorously compare it against other maintenance strategies.

## Data Availability

Data will be available on request.
